# Assessment of clinical handover among ICU nurses based on the structured ISBAR model (2025)

**DOI:** 10.1186/s12912-026-04564-5

**Published:** 2026-04-06

**Authors:** Marzie Yari, Fatemeh Sadat Izadi-Avanji, Mahdieh Sabery

**Affiliations:** Trauma Nursing Research Center, Kashan University of Medical Sciences, Kashan, Iran

**Keywords:** Clinical handover, Patient handover, Clinical handoff, Patient sign out, Intensive care unit

## Abstract

**Background:**

Clinical handover is recognized as a fundamental and important factor in patient safety. About 80% error rate and 70% of serious adverse events related to incomplete clinical handovers. Effective handovers help to reduce medical errors, prevent inappropriate interventions, and ensure continuity of care. In contrast, incomplete handovers may lead to incorrect clinical judgments, delayed treatment, and increased healthcare costs. This study aimed to assess the current status of clinical handover in intensive care units based on the structured ISBAR tool (Identification, Situation, Background, Assessment, Recommendation).

**Methods:**

A descriptive cross-sectional study was conducted in 2025 at six ICUs of Shahid Beheshti Hospital, Kashan, Iran. Using convenience sampling, 124 eligible ICU nurses were included. A total of 370 clinical handovers were directly observed and evaluated. Data were collected via a demographic questionnaire and a structured ICU handover checklist. Descriptive statistics (frequency, percentage, mean, and standard deviation) were analyzed using SPSS version 16.

**Results:**

370 Clinical handovers from 124 nurses were evaluated. Most of the nurses were female (84.7%) with a bachelor’s degree (90.3%) and were employed under permanent contracts (74.2%). The nurses were aged 28 to 48 years, with 4 to 24 years of work experience. Finding revealed low to moderate compliance (21–58%) with the “**Identification”** indicator, moderate to good compliance (67–76%) with “**Situation”** indicator, poor compliance (19.45%) with **“Background**” indicator, a notable variation from poor to excellent compliance (9.2% to 83.19%) for the **“Assessment”** indicator and finally excellent compliance (91–96%) with “**Recommendation**” indicator in clinical handovers among ICU nurses.

**Conclusion:**

This study highlights substantial variability in nurses’ compliance with ISBAR components during ICU clinical handovers. Low to moderate adherence was observed for Identification and Background, with inconsistent performance in Assessment. In contrast, Situation and Recommendation indicators showed considerably higher compliance, reflecting their perceived priority for conveying urgency and actionable care plans. These gaps, likely driven by absent standardized protocols, high workload, time constraints, inadequate training, and assumptions of shared knowledge, increase risks of misidentification, communication breakdowns, and compromised patient safety.

**Clinical trial number:**

Not applicable.

## Background

Clinical handover is critical to patient safety for communicating important patient information from one nurse to the next during shift change [1]. Accurate transfer of information is a fundamental element in making clinical decisions and providing effective, high- quality nursing care [2]. Clinical handover is a key process to improve patient safety. It is the most frequent communication process among nurses in managing patient care. This exchange must present a clear picture of the diagnosis, hemodynamic status, care plan, medical updates, and any other patient-related changes [3]. Effective clinical handover plays a vital role in minimizing errors and ensuring continuity of care in clinical environments [4]. 

Evidence indicates that ineffective clinical handover is recognized as one of the leading causes of adverse events in hospitals worldwide, with 80% of errors throughout the handover process was attributed to poor communication [5] The Patient Safety Committee in the United States has also identified poor information transfer during handover as the primary factor in 65% of irreversible incidents [3]. Incomplete handover can result in inappropriate treatment, delays in diagnosis and therapy, and even increased healthcare costs [6]. Moreover, weak handover practices hinder the prioritization of essential care for patients [2]. It has been reported that about 70% of serious adverse events in hospitals related to insufficient or non-existent handovers [7]. 

Evidence suggests the use of structured, standardized frameworks for clinical handover leads to reduced medical errors, fewer adverse events, enhanced patient safety, and quality of care [4, 8] In Iran, the shift handovers do not have any integrated protocol. shift handovers are usually given verbally using the Kardex. However, the contents of Kardex do not reflect the patient’s caring priorities [9]. One of the effective methods of clinical handover is ISBAR model (standardized framework Identification, Situation, Background, Assessment, Recommendation) which is designed for general wards [10, 11], however, there is no specific tool for Intensive Care Units (ICU) [9]. Given the complex and delicate condition of patients in ICU, it is essential that patient information transfer completely and preciously because even minor errors can lead to irreversible changes and irreparable damages [9, 12]. Evidence showed that effective clinical handover in ICUs resulted in a 75% decrease in pressure ulcers, an 80% decrease in patient falls, and a 50% drop in medication errors [8]. Considering with important issue of clinical handover in ICUs, this study aimed to assess the current status of clinical handover in intensive care units based on the structured ISBAR tool (Identification, Situation, Background, Assessment, Recommendation).

## Methods and materials

### Study design

A descriptive cross-sectional study was conducted in 2025 to identify how nurses conducted clinical handover in ICUs. The research population consisted of all nurses working in six ICUs (two medical ICU, surgical ICU, neurosurgical ICU, pediatric ICU, open heart surgery ICU) in Shahid Beheshti Hospital in Kashan, Iran. Inclusion criteria consisted of all ICU nurses currently working in intensive care units (ICUs), minimum of one year of full-time equivalent clinical experience in ICU settings (to ensure sufficient exposure to the ICU environment and relevant phenomena under study), currently employed in a direct patient-care role in the ICU in three shifts of morning, evening and night. Nurses working exclusively in non-clinical roles (e.g., nurse educators, managers without regular bedside shifts were considered as an exclusion criterion. Convenience sampling method was used. The sample size was determined based on the Scolari study [11] in which the utilization rate of clinical handover based on ISBAR model in ICUs was reported to be 41% and with a 95% confidence level (α = 0.05) and a desired precision of 5% (d = 0.05), the sample size was calculated 370 clinical handovers.

### Data collection

After obtaining ethical approval and permission, the researcher first introduced herself to hospital administrators and explained the objectives of the research. Informed consent was then completed by nurses. As a routine, clinical handover is carried out verbally with the presence of the clinical handover team at the beginning of each shift (morning, evening, and night). The clinical handover team includes all the nurses delivering and receiving the shift and the shift manager or head nurse. The delivering nurse is required to convey the necessary information for her patient to all the nurses and the nurse in charge of the shift during clinical handover. All clinical handovers were observed by first researcher and direct observation during clinical handovers. Three clinical handovers in three shifts of morning, evening, and night for each nurse were directly observed through clinical handovers checklist. That is, each nurse was observed three times (morning shift, evening shift and night shift). As a result, 370 clinical handovers from 124 eligible ICU nurses were observed and evaluated. Data were collected from 1 September (2024) to 11 March (2025) Decembre (about 6 months). Before starting data collection, the first researcher (M.Y) who was nursing student in master science degree of critical care nursing had to present in the environment of ICUs for six months intermittently as an internship course to reduce the Hawthorne effect (where nurses may have altered their behavior knowing they were being observed, potentially leading to an overestimation of handover quality) [13].

### Instruments

A demographic questionnaire and an ICU clinical handover checklist were used for data collection which are both researcher-developed. The demographic information such as age, gender, employment type, shift type (morning, evening, and night), type of working day (week day or weekends), years of work experience, working hours in month were collected with demographic questionnaire.

### ICU clinical handover checklist

The clinical handover checklist was developed by research team for this study. At first, based on literature review and use of structured ISBAR model (Identification, Situation, Background, Assessment, Recommendation) as a base. ISBAR is the standardized framework of clinical handover which is recommended by WHO [10]. This model is used in general wards, however, there is specialized clinical handover tool in ICUs. As a result, a comprehensive item pool was generated with 30 items and underwent psychometric evaluation. The qualitative and quantitative face validity were done with 10 ICU nurses to assess clarity of words in each item (qualitative) and calculate the impact score of each item (quantitative). In qualitative face validity, 3 items were revised and in quantitative face validity, all items obtained an impact score of more than 1.5 and reserved.

In content validity phase, content validity ratio (CVR) and content validity index (CVI) were calculated with expert panel of 10 nursing faculty members in Kashan University of Medical Sciences. All items had CVR greater than 0.62 and reserved. Then, two items with an CVI of less than 0.70 was excluded, and 3 items with an CVI of 0.70–0.79 were revised and a clinical handover checklist with 28 items entered to reliability phase.

To evaluate reliability, two raters (two students in master’s degree) independently observed 20 clinical handovers and completed the ICU clinical handover checklist. The inter-rater agreement coefficient method was used and the weighted kappa coefficient was calculated. The weighted kappa coefficient between observers’ scores ranged from 0.868 to 0.954. Finally, a valid and reliable ICU clinical handover checklist based on structured ISBAR model was developed with 28 items and five indicators— of Identification (3 items), Situation (3 items), Background (1 item), Assessment (18 items), Recommendation and follow up (3 items).

### Scoring of ICU clinical handover checklist

Each item was assessed with a 3-point Likert scale (no, yes and no indication). For scoring, the number " 0” was assigned to the answer “No”, the number " 1” was assigned to the answer “Yes”, and the number “9” was assigned to the answer “No indication”. In the case of “No indication” as a missing data, the percentages were calculated based on “Yes” and “No “answers. Items are not summable in each indicator and in total, and information for each item was reported as frequency and percentage. Adherence to each checklist item was categorized based on the percentage of “Yes” responses among applicable observations (excluding “No indication” cases) as follows:

- ≤ 25%: **poor** (or weak) adherence.
- 26–50%: **moderate** adherence.
- 51–75%: **good** adherence.
- ≥ 75%: **excellent** adherence.

### Data analysis

Data analysis was performed using SPSS version 21 (SPSS Inc., Chicago, IL, USA). Descriptive statistics such as frequency and percentage were presented for qualitative variable (gender, employment type, shift type (morning, evening, and night), type of working day (week day or weekends) and all items of clinical handover checklist (yes, no, no indication). In addition, mean and standard deviation were reported for quantitative variables (age, years of work experience, working hours in month). The significance level was set at 0.05.

## Results

In this study, the majority of nurses were female (84.7%), held a bachelor’s degree in nursing (90.3%), and were employed under permanent contracts (74.2%). The age range of participants was between 28 and 48 years, with work experience ranging from 4 to 24 years (Table 1).

**Table 1:** Demographic information of nurses in the study (*N* = 124)

Qualitative variables		Frequency (%)
Gender	Woman	105(84.7)
	Man	19(15.3)
Education	Bachelor of degree	112(90.3)
	Master of science	12(7.9)
Employment	permanent contracts	92(74.2)
	Contract	17(12.1)
	Probationary	15(13.7)
Shift type	Morning	124(33.5)
	Evening	123(33.2)
	Night	123(33.2)
Type of working day	Week day	104(83.9)
	weekend	20(16.1)
**Quantitative variable**	**Ranges**	**Mean (SD)**
Age	28–48	36.60(4.52)
Working hour at month(hour)	138–310	231.82(46.78)
Working experience(year)	4–24	12.57(5.05)

**Table 2:** Clinical Handover Status of the “**identification**” Indicator the Intensive Care Units (*N* = 370)

ISBAR Indicators			Yes	No	No indication	
			N (%)	N (%)	N (%)	
**Identification**	Item 1	The patient is introduced by full name.	214(57.8)	156(42.2)	-	-
	Item 2	The reason for the patient’s hospitalization is stated.	77(20.8)	293(79.2)	-	-
	Item 3	The attending physician’s name is stated.	96(25.9)	274(74.1)	-	-

Table 2 shows low to moderate compliance (21–58%) with **Identification** indicators in clinical handovers from ICU to ward nurses. It demonstrates relatively moderate compliance with basic patient identification (57.8%) while the low compliance was reported for the hospitalization reason (20.8%) (Table 2).

**Table 3:** Clinical Handover Status of the “**Situation**” Indicator in the Intensive Care units (*N* = 370)

Indicators ISBAR			Yes	No	No indication
			N (%)	N (%)	N (%)
**Situation**	Item 4	The patient’s current overall status is reported (e.g., stable, deteriorating, critical, improving).	282(76.21)	88(23.78)	-
	Item 5	The chief complaint or main problem at the moment is identified and reported (e.g., severe shortness of breath, chest pain, suspected sepsis).	248(67.02)	122(31.97)	-
	Item 6	Immediate life-threatening issues or instability requiring urgent attention are highlighted (e.g., airway compromise, active bleeding, post-resuscitation instability).	136(71.5)	54(28.5)	180*

*Percentages are based on Yes and No answers

Table 3 shows moderate to good compliance (67–76%) with **Situation** indicator in clinical handovers from ICU to ward nurses. The highest adherence was for reporting immediate life-threatening issues (71.5%), followed by overall patient status (76.21%), while explicit identification of the chief complaint was the lowest (67.02%) (Table 3).

**Table 4:** Clinical handover status in the “**Background**” indicator in intensive care units (*N* = 370)

Indicators ISBAR			Yes	No	No indication
			*N* (%)	*N* (%)	*N* (%)
**Background**	Item 7	The patient’s past medical history	72(19.45)	298(80.54)	-

Findings demonstrated that nurses did not report any information about patients ‘past medical history’ in about 80% of handovers and compliance with **Background** indicator was poor (Table 4).

**Table 5:** Clinical handover status in the “**Assessment**” indicator in intensive care units (*N* = 370)

ISBAR Indicators			Yes	No	No indication
			N (%)	N (%)	N (%)
**Assessment**	Item 8	The patient’s level of consciousness is reported using either the Glasgow Coma Scale (GCS) or the FOUR Score (Full Outline of Un Responsiveness) (eye response, motor response, brainstem reflexes, and respiration pattern).	212(57.3)	158(42.7)	-
	Item 9	The abnormal pupillary reactions to light and any changes in the patient’s condition are reported.	212(57.3)	158(42.7)	-
	Item 10	The patient’s hemodynamic status is assessed, and any cases of hemodynamic instability, the interventions performed, and the patient’s response are reported.	291(78.6)	79(21.4)	-
	Item 11	In invasive ventilation, ventilator settings (e.g., mode, FIO₂, etc.) are reported.	168(58.94)	117(41.1)	85*
	Item 12	In non-invasive ventilation (nasal cannula, simple mask, reservoir mask, Venturi mask), the type of oxygen delivery device and the administered oxygen flow rate are specified.	43(50.59)	42(49.41)	285*
	Item 13	It reports the intra-aortic balloon pump (IABP) settings.	48(72.73)	18(27.27)	304*
	Item 14	It reports the type of intravenous fluid, the volume administered, and any adjunct medications added.	189(54.47)	158(45.53)	23*
	Item 15	It reports the time of replacement of patient ‘s connections such as IV sets, central venous catheters, peripheral cannulas, foley catheters, ventilator circuits, heat and moisture exchangers (HME), nasogastric tubes, arterial catheters, and oxygen delivery devices.	34(9.2)	336(90.8)	-
	Item 16	In cases where 24-hour infusion medications are administered, the dosage and any changes in dosage are documented.	225(70.97)	92(29.03)	53*
	Item 17	The type and amount of nutrition are reported.	244(65.9)	126(34.1)	-
	Item 18	the patient’s bowel movements, stool color and consistency are reported.	64(17.3)	306(82.7)	-
	Item 19	Any abnormalities in urinary output and its characteristics are reported.	128(34.6)	242(65.4)	-
	Item 20	The amount and type of abnormal drainage from devices such as nasogastric tubes, colostomies, ileostomies, urostomies, and others are reported.	272(83.19)	55(16.81)	43*
	Item 21	The characteristics of chest tube drainage and indicates whether the device is functioning correctly or not are reported.	130(74.71)	44(25.29)	196*
	Item 22	The laboratory test results and any critical findings are reported.	259(74)	91(26)	20*
	Item 23	**S**ignificant changes in the Morse, Braden, and Wells criteria are reported separately.	81(21.9)	289(78.1)	
	Item 24	Pain management is reported.	150(40.54)	220(59.45)	-
	Item 25	The Richmond Agitation-Sedation Scale (RASS) **i**n agitated patients is assessed and the need for appropriate interventions—such as physical or chemical restraints—is reported.	80(21.62)	305(78.37)	-

The **“Assessment”** indicator was reported in notable variation from poor to excellent compliance (9.2% to 83.19%. The poor compliance was reported for item 15 and the most compliance was associated with item 19. (Table 5)

Table 6 shows excellent compliance (91–96%) with **Recommendation** indicator in clinical handovers (Table 6).

**Table 6:** Clinical handover status in the “**Recommendation**” indicator in intensive care units (*N* = 370)

ISBAR Indicators			Yes	No	No indication
			N (%)	N (%)	N (%)
**Recommendation**	Item 26	The patient’s medical visits and consultations are reported.	355(96.99)	11(3)	4*
	Item 27	The patient’s paraclinical procedures are reported.	295(91.9)	26 (8.1)	49*
	Item 28	The patient’s laboratory tests are reported.	280(95.89)	12(4.11)	78*

*Percentages are based on Yes and No answers

## Discussion

In this study, clinical handovers in intensive care units were examined across five key indicators—Identification, Situation, Background, Assessment, and Recommendations).

In line with one of the primary objectives of this study—to evaluate the level of nurses’ compliance to crucial indicators of clinical handovers in intensive care units—the findings demonstrated that low to moderate compliance (21–58%) with the “Identification”. This level of compliance contrasts sharply with previous research. Pakcheshm et al. (2020) reported an 86.9% adherence rate to the patient identity in coronary care units before ISBAR implementation (rising to 100% afterward) [6], while Kaltoft et al. (2022) documented high adherence to the identity component in postoperative handovers, with related structured-communication metrics around 84% [12]. Similarly, a quality-improvement study on ICU handovers found baseline rates of stating the patient’s name and year of birth at 80%, which increased to 96% and 92%, respectively, following implementation ISBAR tool [14].

This marked discrepancy likely reflects the lack of a standardized handover framework and insufficient emphasis on patient safety culture within the participating healthcare facility. Accurate patient identification constitutes a foundational element of patient safety and serves as a critical safeguard against misidentification errors and associated adverse events [15]. This limited attention arises from inadequate training, high workload, time pressure in the ICU environment, and reliance on unstructured verbal handovers.

The results of this study indicated that nurses’ compliance level for the “Background” indicator during clinical handovers in intensive care units was 19.5%. This low level of adherence is consistent with several studies demonstrating selective or incomplete inclusion of background information in nursing handovers, particularly in ICUs where immediate clinical priorities often dominate. For example, Beigmoradi et al. reported low adherence to clinical background details (10.0%) in general wards [15]. This low rate may be explained by the assumption among nurses that they are already familiar with the patient’s background information, because, ICU nurses often work with the same patients across multiple shifts, which can foster a sense of shared knowledge within the team. Consequently, they may prioritize reporting the patient’s immediate clinical status, vital signs, and ongoing treatments, while omit historical details that are perceived as redundant. Although this assumption may save time in high-pressure environments, it carries the risk of incomplete communication, particularly when new staff members or cross-unit transfers are involved [16]. In contrast, higher compliance level was reported for background component among team leaders (88%) and doctors (83.6%) when ISBAR framework was applied. This higher compliance with the “Background” component appears to have been reported in clinical settings that used structured communication tools [17, 18].

The findings indicated a marked variation in nurses’ compliance to different elements of “Assessment” indicator during clinical handover, ranging from as high as 83.19% (the report of amount and type of abnormal drainage from devices such as nasogastric tubes, colostomies, ileostomies, urostomies) to as low as 9.2% (the report of replacement time of patient ‘s connections). Consistent with this, some studies have revealed that some components of the body system assessment were not consistently addressed and diverse range of information was reported from 22% to 96% [14, 17, 19, 20]. This substantial disparity highlights that nurses prioritize aspects directly tied to immediate or acute patient stability, while longer-term elements or ongoing care receive considerably less emphasis. Such inconsistencies may contribute to gaps in continuity of care, as overlooked details could delay recognition of emerging complications related to body system function or device management. The tendency to deprioritize these elements may stem from the high workload, time constraints and the inherently critical nature of patients in ICUs, where nurses often prioritize immediate life-saving interventions over long term care and consequences [14, 17]. To enhance patient safety and holistic care, a culture of long-term care and use of standardized tools should be incorporated.

The results revealed the Situation-related items and Recommendation-related items received considerable adherence during clinical handover. Consistent with this, A cross-sectional study illustrated that compliance levels between 71.2% and 83.9% for the “Recommendation” component and compliance level between 52.7% to 92.2% for “Situation” component [21]. These components directly address urgency, clarity, and actionable decision-making to enhance patient safety. The Situation component delivers a concise summary of the patient’s current condition and the reason for the communication (e.g., acute deterioration or specific concern), establishing immediate shared situational awareness and signaling priority without extraneous details. The Recommendation component explicitly outlines required actions, requests, or next steps (e.g., urgent review, specific interventions), shifting from descriptive reporting to proactive guidance and ensuring closure on decisions. Evidence from systematic reviews shows that Recommendation reduces hierarchical barriers by encouraging assertive suggestions, facilitates rapid decision-making, lowers medication errors and adverse events, and significantly boosts overall patient safety and care quality [22–24]. Together, these components transform handovers from passive information transfer into targeted, response-oriented exchanges [14]. 

### Limitations

This study has several limitations that warrant cautious interpretation of the findings. First, it was conducted in a single hospital with only six ICUs, limiting the generalizability of results to other settings. Second, convenience sampling of nurses may have introduced selection bias, as participants might differ from experience, motivation, or communication skills, potentially influencing the representativeness of the sample and compliance estimates. Third, direct observation, while objective, is susceptible to the Hawthorne effect. Nurses’ knowledge of being observed could have temporarily improved their performance, despite efforts to minimize this bias, possibly leading to overestimated compliance in some ISBAR components. Fourth, the observation checklist lacked formal assessment of construct validity (e.g., factor analysis), although content validity and inter-rater reliability were established. These limitations highlight the need for future multicenter, longitudinal studies with random or stratified sampling, and broader inclusion of handover stakeholders to provide a more comprehensive understanding of structured handover practices in intensive care settings.

## Conclusion

This study highlights substantial variability in nurses’ compliance with ISBAR components during ICU clinical handovers. Low to moderate adherence was observed for Identification (21–58%) and Background (19.5%), with inconsistent performance in Assessment (9.2%–83.19%). In contrast, Situation and Recommendation elements showed considerably higher compliance, reflecting their perceived priority for conveying urgency and actionable care plans.

These gaps, likely driven by absent standardized protocols, high workload, time constraints, inadequate training, and assumptions of shared knowledge, increase risks of misidentification, communication breakdowns, and compromised patient safety. Current findings underscore the urgent need for structured handover tools, enhanced training, and a stronger patient safety culture in the participating ICU. Adopting such evidence-based interventions is essential to improve consistency, reduce errors, and enhance care continuity in high-acuity settings. For future research, it is recommended to implement a standardized ISBAR checklist in intensive care units and address environmental and workload barriers.

## Data Availability

The datasets used and/or analyzed during the current study are available from the corresponding author on reasonable request.

## Abbreviations

ICU: Intensive Care Unit
ISBAR: Identification, Situation, Background, Assessment, Recommendation
CVR: Content Validity Ratio
CVI: Content Validity Index
FOUR Score: Full Outline of Un Responsiveness (eye response, motor response, brainstem reflexes, and respiration pattern)
GCS: Glasgow Coma Scale
FIO₂: Fraction of inspired oxygen
IABP: Intra-aortic balloon pump
